# Supplementary material for: Protocol for a conversation-based analysis study: PREVENT-ED investigates dialogue features that may help predict dementia onset in later life
Source: BMJ Open. 2019 Mar 27;9(3):e026254. doi: 10.1136/bmjopen-2018-026254 (PMC6475209; doi:10.1136/bmjopen-2018-026254)
Supplement: Author's manuscript [file bmjopen-2018-026254.draft_revisions.pdf]

BMJ Open is committed to open peer review. As part of this commitment we make the peer review history of every article we publish publicly available.

When an article is published we post the peer reviewers' comments and the authors' responses online. We also post the versions of the paper that were used during peer review. These are the versions that the peer review comments apply to.

The versions of the paper that follow are the versions that were submitted during the peer review process. They are not the versions of record or the final published versions. They should not be cited or distributed as the published version of this manuscript.

BMJ Open is an open access journal and the full, final, typeset and author-corrected version of record of the manuscript is available on our site with no access controls, subscription charges or pay-per-view fees (http://bmjopen.bmj.com).

If you have any questions on BMJ Open's open peer review process please email info.bmjopen@bmj.com

BMJ Open

Conversation-based analysis for automatic monitoring of cognitive status: PREVENT-ED investigates dialogue features that may help predict dementia onset in later life

Journal:	*BMJ Open*
Manuscript ID	bmjopen-2018-026254
Article Type:	Protocol
Date Submitted by the Author:	31-Aug-2018
Complete List of Authors:	de la Fuente Garcia, Sofia; University of Edinburgh School of Molecular Genetic and Population Health Sciences, Usher Institute of Population Health Sciences and Informatics<br>Ritchie, Craig; Centre for Clinical Brain Sciences, Department of Psychiatry, University of Edinburgh<br>Luz, Saturnino; University of Edinburgh School of Molecular Genetic and Population Health Sciences, Usher Institute of Population Health Sciences and Informatics
Keywords:	Early detection of Alzheimer's Disease, Dementia prevention, Dialogue analysis, computational paralinguistics, psycholoinguistics, screening

SCHOLARONE™
Manuscripts

Conversation-based analysis for automatic monitoring of cognitive status: PREVENT-ED investigates dialogue features that may help predict dementia onset in later life

Sofia de la Fuente[1], Craig Ritchie[2], Saturnino Luz[1]

1. Edinburgh Medical School: Usher Institute of Population Health Sciences and Informatics. The University of Edinburgh
2. Dementia Prevention Research Group, The University of Edinburgh

Correspondence to: Sofia de la Fuente; sofia.delafuente@ed.ac.uk

For peer review only

Contributions

All authors contributed equally to the study design and writing of the manuscript.

The corresponding author attests that all listed authors meet authorship criteria and that no others meeting the criteria have been omitted.

Funding Statement

This work is supported by the Medical Research Council (MRC), grant number MR/N013166/1.

Competing interest statement

We have read and understood BMJ policy on declaration of interests and declare that we have no competing interests.

Dates of the study:

- Data collection: June 2018 - January 2019.
- Data analysis and dissemination: from February 2019 - end of resources.
- REC approval granted on 01 June 2018 by London-Surrey Research Ethics Committee (IRAS ID: 245117; REC reference No.: 18/LO/0860; Protocol No.: AC18040)
- Caldicott & IG approval granted on 4th June 2018 (Caldicott reference No.: CRD18048)
- R&D approval granted on 7th June 2018 (R&D reference No.: 2018/0125)

Data sharing statement: No additional data are available

For peer review only - http://bmjopen.bmj.com/site/about/guidelines.xhtml

Abstract (250 words structured abstract)

[Introduction]:
Decreasing the incidence of Alzheimer's Disease (AD) is a global public health priority. Early detection of AD is an important requisite for the implementation of prevention strategies towards this goal. While it is plausible that patients at the early stages of AD may exhibit subtle behavioural signs of neurodegeneration, neuropsychological testing seems unable to detect these signs in preclinical AD. Recent studies indicate that spontaneous speech data, which can be collected frequently and naturally, provide good predictors for AD detection in cohorts with a clinical diagnosis. The potential of models based on such data in detecting preclinical AD remains unknown.

[Methods and analysis]:
The PREVENT-ED (PREVENT: Elicitation of Dialogues) study builds on the PREVET Dementia project to investigate whether early behavioural signs of AD may be detected in dialogue interaction. Participants recruited through PREVENT, aged 40-59 at baseline, will be included in this study. We will use speech processing and machine learning methods to assess how well speech and visuospatial markers agree with neuropsychological, biomarker, clinical, lifestyle and genetic data from the PREVENT cohort.

[Ethics and dissemination]:

There are no expected risks or burdens to participants. The procedures are not invasive and do not raise significant ethical issues. We only approach healthy consenting adults and all participants will be informed that this is an exploratory study and therefore has no diagnostic utility. Confidentiality aspects such as data encryption and storage comply with the GDPR and the requirements from sponsoring bodies and ethical committees. Results from this study will be published in peer-reviewed journals.

[Keywords]:
Dementia prevention, early detection of Alzheimer's disease, dialogue analysis, computational paralinguistics, psycholinguistics, screening

For peer review only - http://bmjopen.bmj.com/site/about/guidelines.xhtml

For peer review only

Strengths and Limitations of the Study

Up to five short bullet points, no longer than one sentence each, that relate specifically to the methods.

1. First study to gather spontaneous dialogue data from subjects at risk of AD for predictive modelling;
2. Incorporates elements to analyse spatial navigation abilities, which recent evidence suggests may be useful in detecting pre-clinical AD;
3. Task design balances naturalness and control (it elicits spontaneous dialogues, aiming for external validity, whilst introducing time and topic constrain, aiming for reasonable inter-subject comparisons);
4. Task does not assess the same range of spatial navigation abilities as 3D-based tasks.
5. Recruitment restrictions inherent to the project might hinder our initial sample size target.

For peer review only

For peer review only - http://bmjopen.bmj.com/site/about/guidelines.xhtml

Introduction

The PREVENT Dementia project is a prospective cohort study that aims to identify early signs of dementia. By developing robust disease models for the pre-clinical stages of neurodegeneration and relating these to risk factors and exo-phenotypes [1]. The data comprise family history of dementia, comprehensive neuropsychological assessment, genetic risk profiles, neuroimaging (structural, functional and metabolic), biological markers (CSF, plasma, urine and salivary) and life-style variables. The PREVENT-ED study examines the predictive potential of information extracted from the participant's speech in spontaneous dialogue and assesses its usefulness for screening and future interventional research in terms of those variables.

Recent studies have investigated the use of speech and language analysis as a source of clinical information for monitoring the progress of neurodegenerative diseases [2]. A recent study by Fraser et al. included semantic, syntactic, information content, and acoustic features in a predictive model which obtained 81% accuracy in distinguishing healthy individuals from people with a diagnosis of Alzheimer's dementia [3]. However, this and other studies in this area [4–6] are limited to individuals with a clinical diagnosis of cognitive impairment and therefore offer little insight into the early stages of neurodegeneration. Moreover, they were cross-sectional in design and drew on relatively small data sets. In contrast, our study is designed to collect data from at-risk healthy individuals, longitudinally, in tandem with the phenotypically rich, ongoing PREVENT Dementia study. In addition, while previous work on AD diagnosis based on speech and language has focused on narrative speech, we will focus conversational data from dialogues.

Dialogue involves a broader range of psychological processes than narrative [7]. Recent work has employed conversational speech features such as repairs, repetitions and turn-taking patterns as predictors of AD [4,8–10]. Our study will collect dialogue data from PREVENT Dementia study participants and analyse them in terms of acoustic and dialogical features from voice samples and transcribed recordings to create predictive models.

For peer review only - http://bmjopen.bmj.com/site/about/guidelines.xhtml

PREVENT-ED will also assess spatial navigation abilities. These abilities appear to be a sensitive early cognitive marker of AD [11], and prior research provides evidence for the decline of these abilities in mild cognitive impairment [12]. While still inconclusive, studies of spatial navigation abilities in pre-clinical stages of AD [13], have prompted increased interest in further investigation of how these skills may be affected in the progression of AD [14]. As the PREVENT neuropsychological battery lacks a spatial navigation task [15], the purpose of our experimental design is (primarily) to elicit natural dialogues, and to assess spatial navigation abilities. These aims are complementary, as the dialogues will be elicited through a navigation task, and therefore the analysis of dialogue transcripts can be used as an additional means of assessing spatial navigation abilities.

For peer review only - http://bmjopen.bmj.com/site/about/guidelines.xhtml

Methods and analysis

Objectives

The primary objective of this study is to

1. examine the predictive potential of information extracted from the participant's speech in spontaneous dialogue and its usefulness for screening and interventional research.

Additionally, we aim to

2. identify specific speech and dialogue features that can help predict cognitive decline leading to Alzheimer's Dementia.
3. assess the relationship between such features and certain risk factors found in healthy mid-life participants. Data on these risk factors have been collected by the PREVENT Dementia project and include:
    a. history of parental dementia
    b. ApoE status (presence of ApoE allele ε4 is associated with high risk of dementia) [16]
    c. neuropsychological evaluations (the COGNITO battery [15])
    d. measures of Aβ42 amyloid in plasma and CSF and increases in Tau and pTau (known markers of cognitive decline and AD) [17]
    e. medial temporal lobe atrophy and white matter lesion volume. The medial temporal lobe is an area of the brain known to be shrunken in people with AD [18].
4. assess the possible associations between spatial navigation abilities and the aforementioned risk factors, and
5. assess associations between dialogue features and spatial navigation abilities.

For peer review only - http://bmjopen.bmj.com/site/about/guidelines.xhtml

Participants: sample size and power calculations

Participants are middle-aged healthy volunteers, who were first recruited from the Edinburgh cohort of the PREVENT Dementia study, starting in 2015 [1,15], on the basis of their family history of dementia.

PREVENT-ED is offered to all individuals in the Edinburgh site who have had their baseline assessment and are due their 2-year follow up as well as prospective new participants entering the PREVENT Dementia project. We envisage the total number of participants to be 75, but exact sample size and group allocation will depend upon achievable targets within the ongoing PREVENT Dementia recruitment.

As with most studies based on machine learning methods it is difficult to estimate precisely the optimal sample sizes and measurable effect size. General estimates of sizes for relatively simple classification algorithms such as Euclidean Distance and Fisher Linear Discriminant functions have been placed at $1.2*f$ and $4.0*f$ respectively, where f is the number of features of the dataset, for an expected probability of misclassification (PMC) at most 50% greater than an asymptotic PMC of 0.1 [19] . In past research we have employed feature sets between 62 features (GeMAPS minimalistic parameter set; [20]) and as many as 6373 low-level speech features (prior to feature set reduction). As in this study, we aim to train combinations of weak classifiers, as done in our preliminary tests [6], using a small number of speech features, a sample size of approximately 75 participants should be adequate. However, we aim to collect data from larger numbers of participants, which would allow us to experiment with richer sets of speech features.

For peer review only - http://bmjopen.bmj.com/site/about/guidelines.xhtml

Experimental procedure and design

Edinburgh PREVENT Dementia participants who show an interest in our study will receive an Information Sheet. On attendance, a member of the research team will address any potential queries and take informed consent, prior to the experiment.

Essentially, the procedure for data collection consists of recording conversations. The experimental task designed by our group was inspired by Anderson's et al. Map-Task study, in which "speakers collaborate verbally to reproduce on one participant's map a route printed on the other's" [21]. In Anderson's Map-Task study participants were assigned alternatively the role of "information giver" or "information follower". The former was given a map with a route drawn on it and asked to give instructions to the latter on how to follow this route on their map, which was nearly identical to the former's map, except for the route marking. Our study differs from this design in that our participants will always act as *information givers* (*leader*) while the researcher will take the *follower* role. Further differences in our study are that both maps are identical, and that in our study participants are able to see each other but not each other's maps. Therefore, we will be collecting non-verbal as well as verbal cues that occur in dialogue through the use of audio and video recordings while the participants undertake this map-task.

The purpose of the task is to generate dialogue interactions that are as naturalistic as possible, while still having some control over the settings. This is in line with recent psycholinguistics findings that show how tasks based on maps and games enhance participant engagement and generate spontaneous conversations [22]. The experimental design has two parts that will happen consecutively:

For peer review only - http://bmjopen.bmj.com/site/about/guidelines.xhtml

1. Wayfinding: intended to generate dialogue through a natural interaction between the researcher (*follower*) and the participant (*leader*). They both have a 2-D map of the same land, with 15 landmarks, but the *participant's* map has drawn routes (Figure 1), whereas the *researcher's* does not (Figure 2). Therefore, they need to work together in order to reach mutual understanding and complete a journey through the land, where several alternative paths are possible. A trained researcher will use scripted prompts either querying the participant's choices or providing feedback to create common ground. This part of the task will be audio-visually recorded.

2. Landmark allocation: intended to observe the participant's spatial navigation abilities and their spatial memory (i.e. memory of spatial information, for instance, the layout of the interior of someone's house). After completing the wayfinding task, the participants will receive a version of the map that has neither routes nor landmarks (Figure 3) and be asked to place an "X" at landmark locations on this blank map. Performance is scored in terms of hits and misses on the landmarks they allocate (total: 0-15), as well as the time taken to allocate them. This procedure to assess spatial navigation abilities is based on previous research [13, 23].

For peer review only - http://bmjopen.bmj.com/site/about/guidelines.xhtml

Data management

Conversational data will be recorded by a device developed by our research group specifically for secure collection of speech and video data in healthcare settings, as well as regular close-range microphones. All storage devices are encrypted with state-of-the-art algorithms (AES-256), dialogues will be transcribed, and ID codes assigned so that only de-identified data will be kept indefinitely within the study database. These procedures are in line with the University of Edinburgh data protection policy, which follows the new General Data Protection Regulation (GDPR).

For peer review only

For peer review only - http://bmjopen.bmj.com/site/about/guidelines.xhtml

Analysis

The following features will be extracted from the recorded data:

1. The Geneva minimalistic acoustic parameter set (GeMAPS) for voice research [20]
2. Alignment of prosodic features (pitch, energy) and speech rate.
3. Dialogue structure features (repair, turn-taking patterns, backchannel behaviour, pauses).
4. Different combinations of:
   a. Voice features: F0, spectral flux, ACF, cepstrum, pitch, onset, beats, energy, voice quality, intensity, vocalisation rhythms.
   b. Content features: mood, sentiment analysis, words, lexical and semantic content.
   c. Video features: gesture, gaze, movements.

The spoken dialogue features extracted from the recordings will be regressed over and correlated with data on the neuropsychological evaluations, genetic profiles, biomarkers, neuroimaging, family history and spatial navigation abilities. This will report how much cognitive variance may be explained through these communication patterns, as well as how much they predict participant's level of risk or expression of early disease.

Analytically, we will employ different computational techniques to develop predictions for neurodegenerative decline based on speech features. The research team will look for significant differences and use appropriate statistical tests depending on the variables chosen as predictors. Speech processing and different machine learning methods, from linear generative classifiers to state- of-the-art deep architectures, will be used to model differences between risk groups. This will enable us to identify candidate speech "markers" that could act as early indicators of dementia onset later in life.

For peer review only - http://bmjopen.bmj.com/site/about/guidelines.xhtml

Ethics and dissemination

There are no expected risks or burdens to participants from participating in this study. The procedures do not raise significant ethical issues as they are not invasive and, we only approach healthy consenting adults.

In addition, all participants will be informed that this is an exploratory study and not a diagnostic test. We will assess the extent to which speech "markers" agree with the score of existing markers and therefore the study cannot find more information than those existing markers. In fact, our research question is to what extent this approach would be a good predictor and generate evidence for it.

The main ethical consideration for this study relates to data confidentiality, as it involves collection of audio-visual data, deemed to be identifiable. A discussion with the ethics consultants led us to apply for Caldicott and Information Governance approval and the study complies with the advised requirements regarding data encryption and storage. Also, science and public communications will only include results on analyses undertaken after pre-processing all recordings, ensuring that audio- visual data will never be published or disseminated.

Results from this study will be published in peer-reviewed journals, aiming for an interdisciplinary audience and with a focus on cognitive well-being.

For peer review only - http://bmjopen.bmj.com/site/about/guidelines.xhtml

Conclusions

PREVENT-ED introduces a novel approach to monitoring for early signs of dementia through the analysis of spoken dialogue. While promising results on dialogue analysis have been reported for people with schizophrenia [24–26], research on speech in AD has focused more on narrative speech (monologue), both from transcribed recordings [27–29] and from signal processing of voice samples [6,30,31]. The task introduced in this study aims to elicit dialogue features such as fluency, self- correction, avoidance, pausing behaviour, backchanneling behaviour, question-answering, content and laughter patterns [4,32]. The map-task generates a spontaneous give and take in order to find a common ground for mutual understanding [21]. Even though it is more spontaneous than a structured interview, the content is still constrained enough to allow reasonable comparisons across subjects. In addition, this task will enable us to collect spatial navigation data.

Current evidence is scarce regarding which tests are sensitive enough to detect neurodegeneration at least 25 years before Alzheimer's Dementia is usually diagnosed. The vast majority of studies take place after the onset of Alzheimer's Dementia. As we aim to study its detection at earlier stages of neurodegeneration, the PREVENT Dementia dataset offers an ideal platform for our study to identify new relevant associations. Together with our proposed collection of dialogues, the analysis of PREVENT Dementia data will add speech-based and conversation-based features to the state-of-the-art models for the progression of preclinical neurodegenerative disease.

For peer review only

For peer review only - http://bmjopen.bmj.com/site/about/guidelines.xhtml

Acknowledgements

We thank the PREVENT research team for their help and willingness to welcome us in their project. We also acknowledge Audrey Yeo, from the Edinburgh College of Art, for her contribution to the design of the map and storyboard.

Figure Legends

Figure 1. Wayfinding task: map for the participant (with drawn routes)

Figure 2. Wayfinding task: map for the researcher (without drawn routes)

Figure 3. Landmark allocation task: map for the participant (blank version of the land)

For peer review only

For peer review only - http://bmjopen.bmj.com/site/about/guidelines.xhtml

References

1 Ritchie CW, Ritchie K. The PREVENT study: a prospective cohort study to identify mid-life biomarkers of late-onset Alzheimer's disease. *BMJ Open* 2012;**2**:1–6. doi:10.1136/bmjopen-2012-001893

2 Boschi V, Catricalà E, Consonni M, *et al.* Connected speech in neurodegenerative language disorders: A review. *Front Psychol* 2017;**8**. doi:10.3389/fpsyg.2017.00269

3 Fraser KC, Meltzer JA, Rudzicz F. Linguistic features identify Alzheimer's disease in narrative speech. *J Alzheimer's Dis* 2016;**49**:407–22. doi:10.3233/JAD-150520

4 Watson CM. An analysis of trouble and repair in the natural conversations of people with dementia of the Alzheimer's type. *Aphasiology* 1999;**13**:195–218. doi:10.1080/026870399402181

5 Lopez-de-Ipiña K, Alonso JB, Solé-Casals J, *et al.* On Automatic Diagnosis of Alzheimer's Disease based on Spontaneous Speech Analysis and Emotional Temperature. *Cognit Comput* 2015;**7**:44–55.

6 Meilán JJG, Martínez-Sánchez F, Carro J, *et al.* Speech in alzheimer's disease: Can temporal and acoustic parameters discriminate dementia? *Dement Geriatr Cogn Disord* 2014;**37**:327–34. doi:10.1159/000356726

7 Pickering MJ, Garrod S. Toward a mechanistic psychology of dialogue. *Behav Brain Sci* 2004;**27**. doi:10.1017/S0140525X04000056

8 Mirheidari B, Blackburn D, Reuber M, *et al.* Diagnosing people with dementia using automatic conversation analysis. In: *Proceedings of Interspeech*. San Francisco: 2016. 1220–4.

9 Mirheidari B, Blackburn D, Harkness K, *et al.* An avatar-based system for identifying individuals likely to develop dementia. 2017;:3147–51.

10 Rudzicz F, Chan Currie L, Danks A, *et al.* Automatically Identifying Trouble-indicating Speech Behaviors in Alzheimer's Disease. In: *16th International ACM SIGACCESS Conference on Computers {&} Accessibility*. 2014. 241–2. doi:10.1145/2661334.2661382

11 Maguire EA, Frith CD, Burgess N, *et al.* Knowing Where Things Are: Parahippocampal Involvement in Encoding Object Locations in Virtual Large-Scale Space. *J Cogn Neurosci* 1998;**10**:61–76. doi:10.1162/089892998563789

12 Lithfous S, Dufour A, Després O. Spatial navigation in normal aging and the prodromal stage of Alzheimer's disease: Insights from imaging and behavioral studies. *Ageing Res Rev* 2013;**12**:201–13. doi:10.1016/j.arr.2012.04.007

13 Allison SL, Fagan AM, Morris JC, *et al.* Spatial navigation in preclinical Alzheimer's disease. *J Alzheimer's Dis* 2016;**52**:77–90. doi:10.3233/JAD-150855.Spatial

14 Miniaci MC, De Leonibus E. Missing the egocentric spatial reference: a blank on the map. *F1000Research* 2018;**7**:168. doi:10.12688/f1000research.13675.1

15 Ritchie K, Carrière I, Su L, *et al.* The midlife cognitive profiles of adults at high risk of late-onset Alzheimer's disease: The PREVENT study. *Alzheimer's Dement* 2017;**13**:1089–97. doi:10.1016/j.jalz.2017.02.008

16 Alexopoulos P, Richter-Schmidinger T, Horn M, *et al.* Hippocampal volume differences between healthy young apolipoprotein e ε2 and ε4 carriers. *J Alzheimer's*

For peer review only - http://bmjopen.bmj.com/site/about/guidelines.xhtml

Dis 2011;**26**:207–10. doi:10.3233/JAD-2011-110356

17 Fagan AM, Roe CM, Xiong C, *et al.* Cerebrospinal fluid tau/beta-amyloid(42) ratio as a prediction of cognitive decline in nondemented older adults. *Arch Neurol* 2007;**64**:343–9. doi:10.1001/archneur.64.3.noc60123

18 O'Brien JT, Lloyd A, McKeith I, *et al.* A longitudinal study of hippocampal volume, cortisol levels, and cognition in older depressed subjects. *Am J Psychiatry* 2004;**161**:2081–90. doi:10.1176/appi.ajp.161.11.2081

19 Raudys SJ, Jain AK. Small sample size effects in statistical pattern recognition: recommendations for practitioners. IEEE Trans. Pattern Anal. Mach. Intell. 1991;**13**:252–64. doi:10.1109/34.75512

20 Eyben F, Scherer KR, Schuller BW, *et al.* The Geneva Minimalistic Acoustic Parameter Set (GeMAPS) for Voice Research and Affective Computing. *IEEE Trans Affect Comput* 2016;**7**:190–202. doi:10.1109/TAFFC.2015.2457417

21 Anderson AH, Bader M, Bard EG, *et al.* The HCRC map task corpus. 1991;**34**:351–66.

22 Buxó-Lugo A, Toscano JC, Watson DG. Effects of Participant Engagement on Prosodic Prominence. *Discourse Process* 2018;**55**:305–23. doi:10.1080/0163853X.2016.1240742

23 Iaria G, Palermo L, Committeri G, *et al.* Age differences in the formation and use of cognitive maps. *Behav Brain Res* 2009;**196**:187–91. doi:10.1016/j.bbr.2008.08.040

24 Howes, McCabe R, Purver M. Using Conversation Topics for Predicting Therapy Outcomes in Schizophrenia. *Biomed Inform Insights* 2013;**6**:39. doi:10.4137/BII.S11661

25 McCabe R, Healey PGT, Priebe S, *et al.* Shared understanding in psychiatrist-patient communication: Association with treatment adherence in schizophrenia. *Patient Educ Couns* 2013;**93**:73–9. doi:10.1016/j.pec.2013.05.015

26 Howes C, Purver M, McCabe R, *et al.* Predicting adherence to treatment for schizophrenia from dialogue transcripts. *Proc 13th Annu Meet Spec Interes Gr Discourse Dialogue* 2012;:79–83.http://dl.acm.org/citation.cfm?id=2392814

27 Prud'hommeaux ET, Roark B. Extraction of narrative recall patterns for neuropsychological assessment. In: *INTERSPEECH (Annual Conference of the International Speech Communication Association)*. 2011. 3021–4.

28 Prud'Hommeaux ET, Roark B. Alignment of spoken narratives for automated neuropsychological assessment. *2011 IEEE Work Autom Speech Recognit Understanding, ASRU 2011, Proc* 2011;:484–9. doi:10.1109/ASRU.2011.6163979

29 Roark B, Mithcell M, Hosom J-P, *et al.* Spoken Language Derived Measures for Detecting Mild Cognitive Impairment. *N Engl J Med* 2011;**19**:2081–90. doi:10.1056/NEJMc1108238#SA1

30 Satt A, Sorin A, Toledo-Ronen O, *et al.* Evaluation of speech-based protocol for detection of early-stage dementia. *Proc Annu Conf Int Speech Commun Assoc INTERSPEECH* 2013;:1692–6.

31 Lopez-De-Ipiña K, Alonso JB, Solé-Casals J, *et al.* Alzheimer disease diagnosis based on automatic spontaneous speech analysis. In: *Proceedings of the 4th International Joint Conference on Computational Intelligence*. 2012. 698–705.

For peer review only - http://bmjopen.bmj.com/site/about/guidelines.xhtml

32 Jones D. A family living with Alzheimer's disease: The communicative challenges. *Dementia* 2015;**14**:555–73. doi:10.1177/1471301213502213

For peer review only

For peer review only - http://bmjopen.bmj.com/site/about/guidelines.xhtml

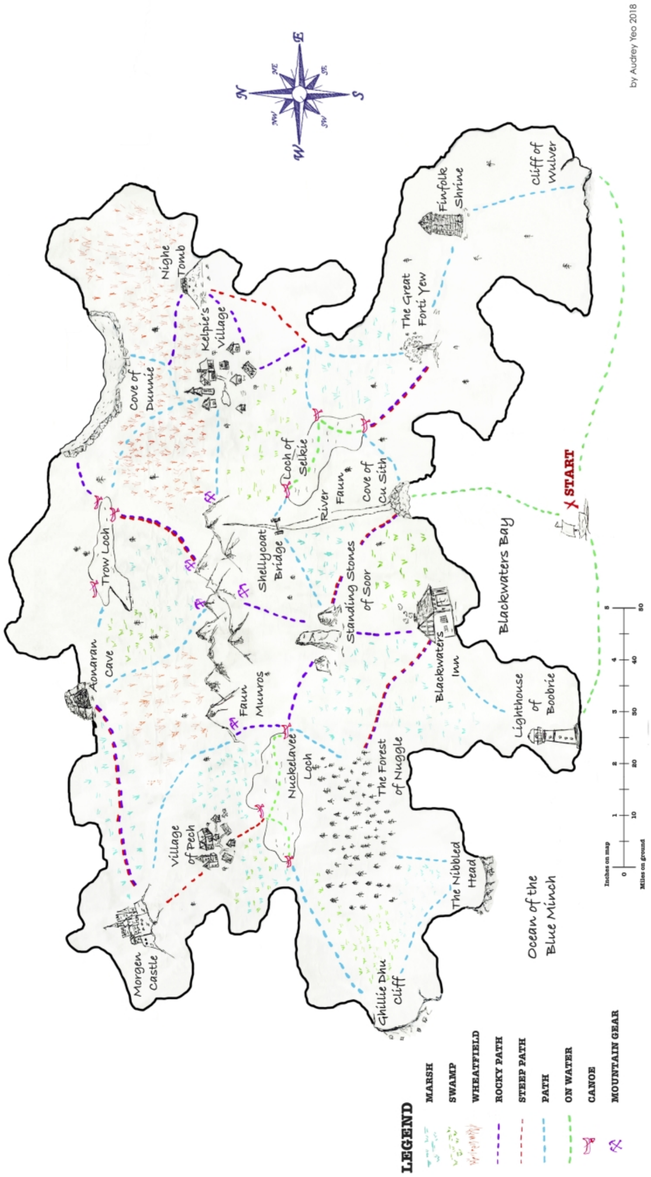

Figure 1. Wayfinding task: map for the participant (with drawn routes)

209x297mm (300 x 300 DPI)

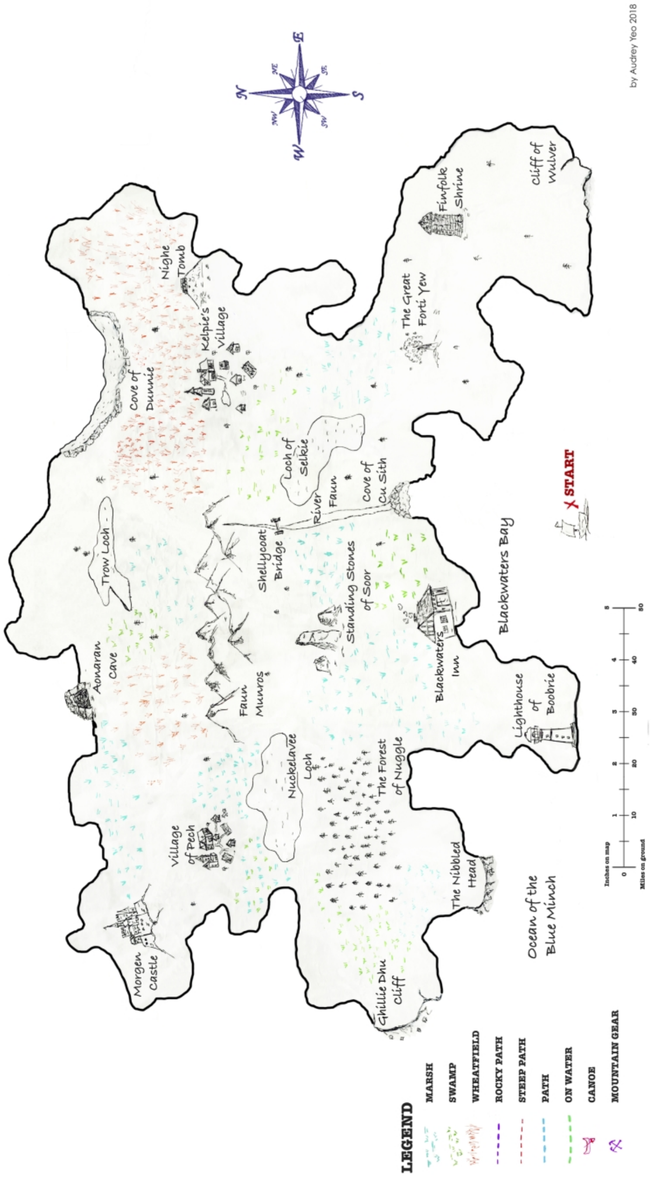

Figure 2. Wayfinding task: map for the researcher (without drawn routes)

209x297mm (300 x 300 DPI)

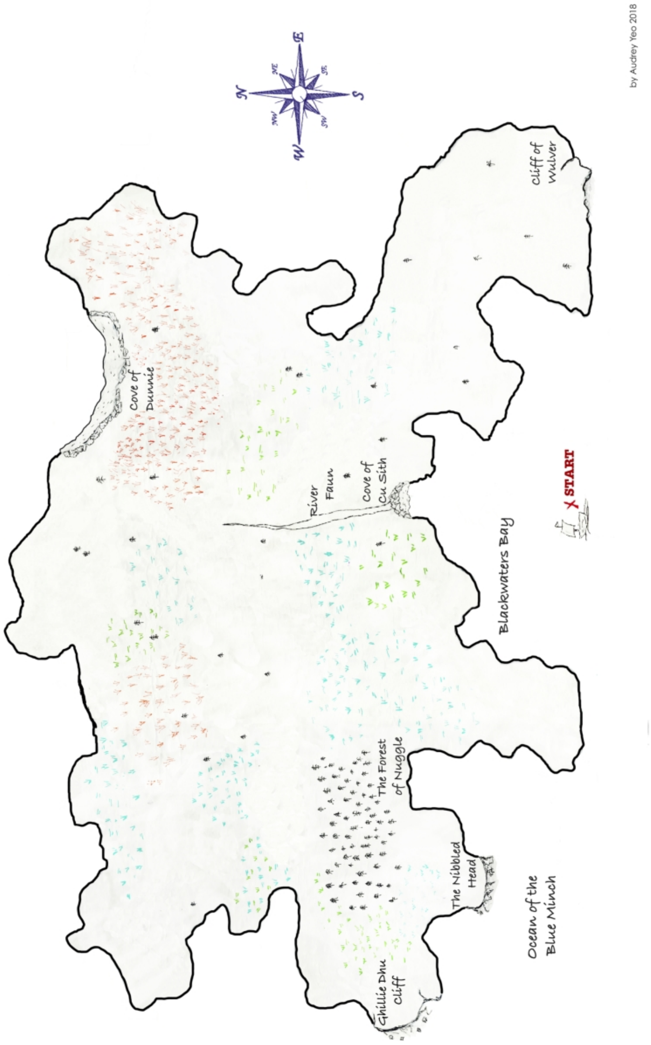

Figure 3. Landmark allocation task: map for the participant (blank version)

209x297mm (300 x 300 DPI)

BMJ Open

Protocol for a conversation-based analysis study: PREVENT-ED investigates dialogue features that may help predict dementia onset in later life

Journal:	*BMJ Open*
Manuscript ID	bmjopen-2018-026254.R1
Article Type:	Protocol
Date Submitted by the Author:	10-Jan-2019
Complete List of Authors:	de la Fuente Garcia, Sofia; University of Edinburgh School of Molecular Genetic and Population Health Sciences, Usher Institute of Population Health Sciences and Informatics<br>Ritchie, Craig; Centre for Clinical Brain Sciences, Department of Psychiatry, University of Edinburgh<br>Luz, Saturnino; University of Edinburgh School of Molecular Genetic and Population Health Sciences, Usher Institute of Population Health Sciences and Informatics
**Primary Subject Heading**:	Health informatics
Secondary Subject Heading:	Communication, Mental health, Research methods, Diagnostics, Geriatric medicine
Keywords:	Early detection of Alzheimer's Disease, Dementia prevention, Dialogue analysis, computational paralinguistics, psycholoinguistics, screening

SCHOLARONE™
Manuscripts

Protocol for a conversation-based analysis study: PREVENT-ED investigates dialogue features that may help predict dementia onset in later life

Sofia de la Fuente[1], Craig Ritchie[2], Saturnino Luz[1]

1. Edinburgh Medical School: Usher Institute of Population Health Sciences and Informatics. The University of Edinburgh
2. Dementia Prevention Research Group, The University of Edinburgh

Correspondence to: Sofia de la Fuente; sofia.delafuente@ed.ac.uk

Contributions

SF co-designed the experiment, elaborated the map and the map task's narrative, wrote the initial draft of the paper and revised it following revision and input from co-authors. CR helped design the experiment, revised and provided feedback on the text, and co-supervised the work. SL conceived the idea of using a dialogue task for cognitive state assessment, co-designed the experiment, wrote and revised the text, and co-supervised the work. All authors read and approved the final version of the paper.

The corresponding author attests that all listed authors meet authorship criteria and that no others meeting the criteria have been omitted.

Funding Statement

This work is supported by the Medical Research Council (MRC), grant number MR/N013166/1.

Competing interest statemen

We have read and understood BMJ policy on declaration of interests and declare that we have no competing interests.

Dates of the study:

- Data collection: August 2018 - May 2019.
- Data analysis and dissemination: from February 2019 - end of resources.
- REC approval granted on 01 June 2018 by London-Surrey Research Ethics Committee (IRAS ID: 245117; REC reference No.: 18/LO/0860; Protocol No.: AC18040)
- Caldicott & IG approval granted on 4th June 2018 (Caldicott reference No.: CRD18048)
- R&D approval granted on 7th June 2018 (R&D reference No.: 2018/0125)

Data sharing statement: No additional data are available

For peer review only

Abstract (250 words structured abstract)

[Introduction]:
Decreasing the incidence of Alzheimer's Disease (AD) is a global public health priority. Early detection of AD is an important requisite for the implementation of prevention strategies towards this goal. While it is plausible that patients at the early stages of AD may exhibit subtle behavioural signs of neurodegeneration, neuropsychological testing seems unable to detect these signs in preclinical AD. Recent studies indicate that spontaneous speech data, which can be collected frequently and naturally, provide good predictors for AD detection in cohorts with a clinical diagnosis. The potential of models based on such data in detecting preclinical AD remains unknown.

[Methods and analysis]:
The PREVENT-ED (PREVENT: Elicitation of Dialogues) study builds on the PREVENT Dementia project to investigate whether early behavioural signs of AD may be detected in dialogue interaction. Participants recruited through PREVENT, aged 40-59 at baseline, will be included in this study. We will use speech processing and machine learning methods to assess how well speech and visuospatial markers agree with neuropsychological, biomarker, clinical, lifestyle and genetic data from the PREVENT cohort.

[Ethics and dissemination]:

There are no expected risks or burdens to participants. The procedures are not invasive and do not raise significant ethical issues. We only approach healthy consenting adults and all participants will be informed that this is an exploratory study and therefore has no diagnostic utility. Confidentiality aspects such as data encryption and storage comply with the GDPR and the requirements from sponsoring bodies and ethical committees. This study has been granted approval by the London-Surrey Research Ethics Committee (REC reference No.: 18/LO/0860), and by Caldicott & Information Governance (reference No.: CRD18048). PREVENT-ED results will be published in peer-reviewed journals.

[Keywords]:
Dementia prevention, early detection of Alzheimer's disease, dialogue analysis, computational paralinguistics, psycholinguistics, screening

For peer review only

Strengths and Limitations of the Study

Up to five short bullet points, no longer than one sentence each, that relate specifically to the methods.

1. First study to gather spontaneous dialogue data from subjects at risk of AD for predictive modelling;
2. Incorporates elements to analyse spatial navigation abilities, which recent evidence suggests may be useful in detecting pre-clinical AD;
3. Task design balances naturalness and control (it elicits spontaneous dialogues, aiming for external validity, whilst introducing time and topic constrain, aiming for reasonable inter-subject comparisons);
4. Task does not assess the same range of spatial navigation abilities as 3D-based tasks.
5. Recruitment restrictions inherent to the project might hinder our initial sample size target.

For peer review only

Introduction

The PREVENT Dementia project is a prospective cohort study that aims to identify early signs of dementia. By developing robust disease models for the pre-clinical stages of neurodegeneration and relating these to risk factors and exo-phenotypes [1]. The data comprise family history of dementia, comprehensive neuropsychological assessment, genetic risk profiles, neuroimaging (structural, functional and metabolic), biological markers (CSF, plasma, urine and salivary) and life-style variables. The PREVENT-ED study examines the predictive potential of information extracted from the participant's speech in spontaneous dialogue and assesses its usefulness for screening in terms of those variables.

Recent studies have investigated the use of speech and language analysis as a source of clinical information for monitoring the progress of neurodegenerative diseases [2]. A recent study by Fraser et al. included semantic, syntactic, information content, and acoustic features in a predictive model which obtained 81% accuracy in distinguishing healthy individuals from people with a diagnosis of Alzheimer's dementia [3]. However, this and other studies in this area [4–6] are limited to individuals with a clinical diagnosis of cognitive impairment and therefore offer little insight into the early stages of neurodegeneration. Moreover, they were cross-sectional in design and drew on relatively small data sets. In contrast, our study is designed to collect data from at-risk healthy individuals, longitudinally, in tandem with the phenotypically rich, ongoing PREVENT Dementia study. In addition, while previous work on AD diagnosis based on speech and language has focused on narrative speech monologues (i.e. most cases are descriptions of a scene such as the Boston "cookie theft" picture description task [7]), we will focus conversational data from dialogues.

Dialogue involves a broader range of psychological processes than monologue. This is because in order to achieve successful communication, speakers need to find a common ground for understanding, which demands coordination and implies alignment and entailment at different levels [8]. Recent work has employed conversational speech features such as repairs, repetitions and turn-taking patterns as predictors of AD [4,9–11]. Our study will collect dialogue data from PREVENT Dementia

For peer review only

study participants and analyse them in terms of acoustic and dialogical features from voice samples and transcribed recordings to create predictive models.

For peer review only

PREVENT-ED will also assess spatial navigation abilities. These abilities appear to be a sensitive early cognitive marker of AD [12], and prior research provides evidence for the decline of these abilities in mild cognitive impairment [13]. While still inconclusive, studies of spatial navigation abilities in pre-clinical stages of AD [14] have prompted increased interest in further investigation of how these skills may be affected in the progression of AD [15]. As the PREVENT neuropsychological battery lacks a spatial navigation task [16], the purpose of our experimental design is (primarily) to elicit natural dialogues, and to assess spatial navigation abilities. These aims are complementary, as the dialogues will be elicited through a discussion over a map-based task, and therefore the analysis of dialogue transcripts can be used as an additional means of assessing spatial navigation abilities.

For peer review only

Methods and analysis

Objectives

The primary objective of this study is to

1. examine the predictive potential of information extracted from the participant's speech in spontaneous dialogue and its usefulness for screening.

Additionally, we aim to

2. identify specific speech and dialogue features that can help predict cognitive decline leading to Alzheimer's Dementia.
3. assess the relationship between such features and certain risk factors found in healthy mid- life participants. Data on these risk factors have been collected by the PREVENT Dementia project and include:
    a. history of parental dementia
    b. ApoE status (presence of ApoE allele ε4 is associated with high risk of dementia) [17]
    c. neuropsychological evaluations with the COGNITO battery [16]. The COGNITO test battery has been developed specifically to look across numerous cognitive domains with tests that are not subject to the ceiling effects of tests designed for use in dementia. It is entirely computer based and has been used in numerous conditions to assess cognition including depression, schizophrenia, The interrater reliability of this battery has been stablished and compared to other cognitive measurements [18] and it has now been translated into five languages and Chinese underway.
    d. measures of Aβ42 amyloid in plasma and CSF and increases in Tau and pTau (known markers of cognitive decline and AD) [19]
    e. medial temporal lobe atrophy and white matter lesion volume. The medial temporal lobe is an area of the brain known to be shrunken in people with AD [20].

For peer review only

4. assess the possible associations between spatial navigation abilities and the aforementioned risk factors, and

5. assess associations between dialogue features and spatial navigation abilities.

For peer review only

Participants: sample size and power calculations

Participants are middle-aged healthy volunteers, who were first recruited from the Edinburgh cohort of the PREVENT Dementia study, starting in 2015 [1,16], on the basis of their family history of dementia.

PREVENT-ED is offered to all individuals in the Edinburgh site who have had their baseline assessment and are due their 2-year follow up as well as prospective new participants entering the PREVENT Dementia project. Hence, if a participant suits PREVENT's inclusion criteria it will also be recruited for PREVENT-ED without further criteria checks, as long as they agree to it (for more information on PREVENT's exclusion and inclusion criteria, please refer to [1] and [16]. The participant's risk status with regards to the factors listed above will remain unknown to the PREVENT-ED researchers at the time of the assessment, in order to avoid potential experimentation biases. These data will have been recorded by the main PREVENT project on separate assessment dates and will be disclosed to PREVENT-ED researchers when the project reaches its data analysis stage.

In terms of the number of participants required, a distinction must be made between the primary and the secondary objectives of the study. The secondary objectives involve hypothesis testing, while the primary objective concerns the creation of machine learning models for prediction. While determination of sample sizes is relatively straightforward for the former, it is less so for the latter. Assessment of the hypothesis that spatial navigation abilities differ in neutral, low and high-risk participants will be done through analysis of covariance, taking the score in the spatial navigation task as the dependent variable. Allison et al. [14] report a large effect size (partial $\eta^2 = 0.564$) for a similar wayfinding task comparing three groups (participants with negative biomarkers). Therefore, conservatively assuming that we wish to be able to detect a medium effect size of 0.16, with a power of 80% at a significance level $p < 0.05$, we require a minimum of 63 participants. As regards the machine learning modelling objective, as with most studies involving automatic categorisation it is difficult to estimate precisely the optimal sample sizes and measurable effect sizes. A widely adopted method of sample size estimation for relatively simple machine learning algorithms such as Euclidean Distance and Fisher Linear Discriminant functions places lower bounds at $1.2 \times f$ and $1.4 \times f$ instances (participants) respectively, where f is the

number of features of the dataset, for an expected probability of misclassification (PMC) at most 50% greater than an asymptotic PMC of 0.1 [21]. In past research we have employed feature sets containing between 62 features (GeMAPS minimalistic parameter set; [22]) and as many as 6,373 low-level speech features (prior to feature set reduction) for similar classification tasks. A motivation for using the GeMAPS feature set is that it will allow comparability with other studies and future replicability, since this is a standardised feature set that has been used in several computational paralinguistics and psycholinguistics tasks, such as affective computing and mood analysis. As we have done in previous work [6], we aim to train combinations of weak classifiers using a small number of speech features, including turn-taking, pause patterns, speech rate, voice energy, and voice quality measures. Therefore, using the GeMAPS feature set in a similar manner implies that a minimum of 75 participants will be necessary for a PMC at most 50% greater than a conservative asymptotic PMC of 0.1 (90% accuracy). However, we aim to collect data from larger numbers of participants, which will allow us to experiment with larger sets of speech features.

For peer review only

Experimental procedure and design

Edinburgh PREVENT Dementia participants who show an interest in our study will receive an Information Sheet. On attendance, a member of the research team will address any potential queries and take informed consent, prior to the experiment.

Essentially, the procedure for data collection consists of recording conversations. The experimental task designed by our group was inspired by Anderson's et al. Map-Task study, in which "speakers collaborate verbally to reproduce on one participant's map a route printed on the other's" [23]. In Anderson's Map-Task study participants were assigned alternatively the role of "information giver" or "information follower". The former was given a map with a route drawn on it and asked to give instructions to the latter on how to follow this route on their map, which was nearly identical to the former's map, except for the route marking. Our study differs from this design in that our participants will always act as *information givers* (*leader*) while the researcher will take the *follower* role. The rationale for this is to control for the potential influence of the role (giver vs. follower) on the strategy participants employ when performing the task. In addition, holding the role of the participant fixed will ensure a level of consistency across the data helping make our conclusions more robust for this particular experiment. Further differences between the PREVENT-ED and the original Map Task study are that in PREVENT-ED both maps are identical, and that participants are able to see each other but not each other's maps. Therefore, we will be collecting non-verbal as well as verbal cues that occur in dialogue through the use of audio recordings while the participants undertake this map-task.

The purpose of the task is to generate dialogue interactions that are as naturalistic as possible, while still having some control over the settings. Although there is a trade-off between control and naturalness, this study design focuses on dialogue interaction mechanisms, rather than dialogue content. Whilst other tasks, such as structured conversations, may elicit more naturally generated content, we would not expect such tasks to generate naturalistic dialogue interaction structures because generally the interviewee would be prompted to speak rather than spontaneously engage in turn-taking, pausing and other interactional behaviours. Thus, the imaginary land to be navigated by the participants is not designed to be a demanding cognitive task, but rather it is designed to generate a cooperative storytelling

For peer review only

and to enhance engagement through the completion of a creative journey. This is in line with recent psycholinguistics findings that show how tasks based on maps and games enhance participant engagement and generate spontaneous conversations [24]. The experimental design has two parts which will happen consecutively:

For peer review only

1. Wayfinding: intended to generate dialogue through a natural interaction between the researcher (*follower*) and the participant (*leader*). They both have a 2-D map of the same land, with 15 landmarks, but the *participant's* map has drawn routes (Figure 1), whereas the *researcher's* does not (Figure 2). Therefore, they need to work together in order to reach mutual understanding and complete a journey through the land, where several alternative paths are possible. The task requires going through different landmarks and trading for certain items at each of them. A trained researcher will use scripted prompts either querying the participant's choices or providing feedback to create common ground. This part of the task will be audio-visually recorded. The rational for the use of this task lies on the aim for eliciting dialogue in a way that resembles a natural setting. Giving and taking directions is an activity that belongs to routine life as much as to the experimental set up, which is nevertheless still a controlled setting in that the follower is a trained researcher who will ensure the participant goes over all necessary landmarks without missing any of them.

2. Landmark allocation: intended to observe the participant's spatial navigation abilities and their spatial memory (i.e. memory of spatial information, for instance, the layout of the interior of someone's house). After completing the wayfinding task, the participants will receive a version of the map that has neither routes nor landmarks (Figure 3) and be asked to place an "X" at landmark locations on this blank map. Performance is scored in terms of hits and misses on the landmarks they allocate (total: 0-15), as well as the time taken to allocate them. The hit and miss scoring procedure consists on:
    a. scoring 1 point for each correct landmark: following the nearest neighbour criteria, that is, 1 point will be awarded if the participant's choice is closer to the target landmark than to other landmarks, regardless of whether it place on the exact spot
    b. subtracting 1 point for each missing landmark: if the participant placed less than 15 landmarks
    c. subtracting 1 point for each misplaced landmark: if a landmark is placed in a different location

d. subtracting 0.5 points for "made-up" landmarks: the participant places more than one landmark where there should be only one, and/or places more than 15 landmarks in total.

This task will always be scored by the same researcher, will follow these criteria according with the research training, undertaken prior to data collection. This procedure to assess spatial navigation abilities is based on previous research [14,25].

During the wayfinding task, the participant does not need to keep in mind the landmarks to be covered on a given route. They only need to focus on giving information about the available paths, specifying terrain conditions, and discussing distances and choices for directions with the researcher, who is the one narrating the journey - that is, signalling which landmark needs to be reached at each stage, and in what order. The journey goes through the same landmarks, in the same order, for all participants, and therefore its potential confounding effect during the analysis of the second task is controlled by it being fixed on the design.

The experimental procedure was tested twice, with two different participants, one involved with the main PREVENT project as a research assistant, and another totally unrelated to this research. These preparatory sessions were successful and helped optimise the experimental setup They led to the choice of A2 (420x594mm) as the size of the printed maps, as well as other settings such as lighting, table height, and recorder placement. These sessions also informed logistic decisions such as setting reasonable time slots for each participant to come for the assessment.

For peer review only

Data management

Conversational data will be recorded by a device developed by our research group specifically for secure collection of speech and video data in healthcare settings, as well as regular close-range microphones. All storage devices are encrypted with state-of-the-art algorithms (AES-256), dialogues will be transcribed, and ID codes assigned so that only de-identified data will be kept indefinitely within the study database. These procedures are in line with the University of Edinburgh data protection policy, which follows the new General Data Protection Regulation (GDPR).

There are two main aspects to the data pre-processing: pre-processing for acoustic analysis and pre-processing for natural language processing. For the acoustic analysis, the ELAN[1] tool [26,27] will be used for transcription and annotation of dialogue events such as speaker turns, false starts – potentially signalling self- repair events – etc. ELAN is a professional tool developed by the Max Planck Institute for Psycholinguistics, designed for complex annotations on audio and video resources. Audio streams may be studied through different perspectives, from low-level features (e.g. frequencies, energy) to medium-level features (e.g. syntax, lexicon) or high-level features (e.g. sentiment analysis). ELAN allows for hierarchically interconnected annotation tiers which make structured annotations possible in such differentiated levels of analysis. For the natural language processing, ELAN will be used, in transcription mode, with which hierarchical annotations can be synchronised (time-stamped and time-aligned). Annotations are stored in XML format. Annotations will also be exported to CHAT, Praat and csv text formats for compatibility with other systems and tools [27].

Patient and Public Involvement Statement

As its participants form a subcohort of the PREVENT study's cohort, PREVENT-ED benefited from the Patient and Public involvement measures already place for that study. The PREVENT Dementia project counts with a Participants' Panel, established in 2013 prior to the development of the whole research programme. This panel is made up of a group of volunteers who are taking part in the study themselves

[1] https://tla.mpi.nl/tools/tla-tools/elan/

For peer review only

and who meet regularly to discuss the progress of the study and the potential addition of sub-studies to the project. Two members of this panel also sit in the Steering Committee. As an additional sub-study, PREVENT-ED proposal was presented to this Participants' Panel, as well as to the Prevent Steering Committee. The potential burdens of the intervention and the time required to participate in the research were assessed and it was decided for the project to be taken forward.

With regards to results disclosure, the procedure within Prevent Dementia is that if something is found which is clinically relevant, it will be fed back to the participant and their GP. This applies to blood tests, ECG, MRI and validated cognitive tests. However, this does not apply to assessments only carried out for research purposes, as is the case of PREVENT-ED, which will not directly yield clinically relevant results.

For peer review only

Analysis

The following features will be extracted from the recorded data:

1. The Geneva minimalistic acoustic parameter set (GeMAPS) for voice research [22]
2. Alignment of prosodic features (pitch, energy) and speech rate. Alignment in this context means convergence to the same rates for certain speech features. It refers to the psycholinguistic theory that assumes that dialogue processes lead to the automatic coupling of linguistic representations between production and comprehension. This occurs on different levels, and implies accommodation, where the speakers attune to each other throughout the conversation [8].

3. Dialogue structure features (repair, turn-taking patterns, backchannel behaviour, pauses), to be extracted from ELAN annotations and tiers [27].

4. Different combinations of:
    a. Voice features: F0, spectral flux, ACF, cepstrum, pitch, onset, beats, energy, voice quality, intensity, vocalisation rhythms.
    b. Content features: mood, sentiment analysis, words, lexical and semantic content.

The spoken dialogue features extracted from the recordings will be regressed over and correlated with data on the neuropsychological evaluations, genetic profiles, biomarkers (amyloid, tau and phosphorylated tau levels in cerebrospinal fluid and plaque-dependant inflammation, cortisol levels), neuroimaging (level of brain atrophy in the medial temporal lobe, particularly the hippocampus and entorhinal cortex) , family history and spatial navigation abilities [1,16]. In other words, a range of techniques will be applied to investigate whether linguistic, dialogical and paralinguistic features are predictive of, or correlate with:

- quantitative scores from Prevent neuropsychological evaluations (COGNITO battery [16])
- categories of ApoE status (presence of ApoE allele ε4 is associated with high risk of dementia) [17]
- categories of history of parental dementia (0, 1 or 2 parents with a dementia diagnosis)

For peer review only

- quantitative measures of Aβ42 amyloid in plasma and CSF and increases in Tau and pTau (known markers of cognitive decline and AD) [19]
- quantitative measures of medial temporal lobe atrophy and white matter lesion volume. The medial temporal lobe is an area of the brain known to be shrunken in people with AD [20].

Pearson bivariate and multivariate tests will be used to assess simple correlations, and predictive Gaussian process regression will be employed for predictive modelling. This will assess how much cognitive variance may be explained through these communication patterns, as well as how much they predict each participant's level of risk or early signs of the disease.

Analytically, we will employ different computational techniques to develop predictions for neurodegenerative decline based on speech features and language. The research team will look for significant differences and use appropriate statistical tests depending on the variables chosen as predictors. Speech signal processing and different machine learning methods, from linear generative classifiers to state- of-the-art deep architectures, will be used to model differences between risk groups. Furthermore, assessment will be ongoing as PREVENT participants are scheduled for a follow-up after at least 2 years and 5 years (with longer term follow-up timespans to be decided). Hence, longitudinal data will eventually be available, including variable outcomes and endpoints where applicable. This will enable us to identify candidate speech markers that could act as early indicators of dementia onset later in life.

For peer review only

Ethics and dissemination

There are no expected risks or burdens to participants from participating in this study. The procedures do not raise significant ethical issues as they are not invasive and, we only approach healthy consenting adults.

In addition, all participants will be informed that this is an exploratory study and not a diagnostic test. We will assess the extent to which speech "markers" agree with the score of existing markers and therefore the study cannot find more information than those existing markers. In fact, our research question is to what extent this approach would be a good predictor and generate evidence for it.

The main ethical consideration for this study relates to data confidentiality, as it involves collection of audio-visual data, deemed to be identifiable. A discussion with the ethics consultants led us to apply for Caldicott and Information Governance approval and the study complies with the advised requirements regarding data encryption and storage. Also, science and public communications will only include results on analyses undertaken after pre-processing all recordings, ensuring that audio-visual data will never be published or disseminated. This study has been granted approval by the London-Surrey Research Ethics Committee (REC reference No.: 18/LO/0860), and by Caldicott & Information Governance (reference No.: CRD18048).

Results from PREVENT-ED will be published in peer-reviewed journals, aiming for an interdisciplinary audience and with a focus on cognitive well-being.

For peer review only

Conclusions

PREVENT-ED introduces a novel approach to monitoring for early signs of dementia through the analysis of spoken dialogue. While promising results on dialogue analysis have been reported for people with schizophrenia [28–30], research on speech in AD has focused more on narrative speech (monologue), both from transcribed recordings [31–33] and from signal processing of voice samples [34–36]. The task introduced in this study aims to elicit dialogue features such as fluency, self-correction, avoidance, pausing behaviour, backchanneling behaviour, question-answering, content and laughter patterns [4,37]. These features will be extracted from annotations and transcripts, as well as from automatically generated GeMAPS data sets, and will be used for machine learning and statistical analysis to explore their relationship with other risk factors for AD, and thus their potential to predict pre-clinical stages of the disease. The map task generates a spontaneous give and take in order to find a common ground for mutual understanding [23]. Even though this setting is more spontaneous than a structured interview, the content is still constrained enough so that consistency across data is expected, allowing for comparisons across subjects. In addition, this task will enable us to collect spatial navigation data, which will be investigated along with the dialogue data.

Current evidence is scarce regarding which tests are sensitive enough to detect neurodegeneration at least 25 years before Alzheimer's Dementia is usually diagnosed. The vast majority of studies take place after the onset of Alzheimer's Dementia. As we aim to study its detection at earlier stages of neurodegeneration, the PREVENT Dementia dataset offers an ideal platform for our study to identify new relevant associations. Together with our proposed collection of dialogues, the longitudinal analysis of PREVENT Dementia data will add speech-based and conversation-based features to the state-of-the- art models for the progression of preclinical neurodegenerative disease.

For peer review only

Acknowledgements

We thank the PREVENT research team for their help and willingness to welcome us in their project, in particular Sarah Gregory, Katie Wells, Clare Dolan and Neil Fullerton. We also acknowledge Audrey Yeo, from the Edinburgh College of Art, for her contribution to the design of the map and storyboard.

Figure Legends

Figure 1. Wayfinding task: map for the participant (with drawn routes)

Figure 2. Wayfinding task: map for the researcher (without drawn routes)

Figure 3. Landmark allocation task: map for the participant (blank version of the land)

For peer review only

References

1 Ritchie CW, Ritchie K. The PREVENT study: a prospective cohort study to identify mid-life biomarkers of late-onset Alzheimer's disease. *BMJ Open* 2012;**2**:1–6. doi:10.1136/bmjopen-2012-001893

2 Boschi V, Catricalà E, Consonni M, *et al.* Connected speech in neurodegenerative language disorders: A review. *Front Psychol* 2017;**8**. doi:10.3389/fpsyg.2017.00269

3 Fraser KC, Meltzer JA, Rudzicz F. Linguistic features identify Alzheimer's disease in narrative speech. *J Alzheimer's Dis* 2015;**49**:407–22. doi:10.3233/JAD-150520

4 Watson CM. An analysis of trouble and repair in the natural conversations of people with dementia of the Alzheimer's type. *Aphasiology* 1999;**13**:195–218. doi:10.1080/026870399402181

5 Lopez-de-Ipiña K, Alonso J., Solé-Casals J, *et al.* On Automatic Diagnosis of Alzheimer's Disease based on Spontaneous Speech Analysis and Emotional Temperature. *Cognit Comput* 2015;**7**:44–55.

6 Luz S, Fuente S De, Albert P. A Method for Analysis of Patient Speech in Dialogue for Dementia Detection. In: *Resources and Processing of linguistic, para-linguistic and extra-linguistic Data from people with various forms of cognitive impairment*. European Language Resources Association (ELRA), Paris, France, pp. 35-42

7 Goodglass H, Kaplan E. The assessment of aphasia and related disorders. 1983.

8 Pickering MJ, Garrod S. Toward a mechanistic psychology of dialogue. *Behav Brain Sci* 2004;**27**. doi:10.1017/S0140525X04000056

9 Mirheidari B, Blackburn D, Reuber M, *et al.* Diagnosing people with dementia using automatic conversation analysis. In: *Proceedings of Interspeech*. San Francisco: 2016. 1220–4.

10 Mirheidari B, Blackburn D, Harkness K, *et al.* An avatar-based system for identifying individuals likely to develop dementia. 2017;:3147–51.

11 Rudzicz F, Chan Currie L, Danks A, *et al.* Automatically Identifying Trouble-indicating Speech Behaviors in Alzheimer's Disease. In: *16th International ACM SIGACCESS Conference on Computers {&} Accessibility*. 2014. 241–2. doi:10.1145/2661334.2661382

12 Maguire EA, Frith CD, Burgess N, *et al.* Knowing Where Things Are: Parahippocampal Involvement in Encoding Object Locations in Virtual Large-Scale Space. *J Cogn Neurosci* 1998;**10**:61–76. doi:10.1162/089892998563789

13 Lithfous S, Dufour A, Després O. Spatial navigation in normal aging and the prodromal stage of Alzheimer's disease: Insights from imaging and behavioral studies. *Ageing Res Rev* 2013;**12**:201–13. doi:10.1016/j.arr.2012.04.007

14 Allison SL, Fagan AM, Morris JC, *et al.* Spatial navigation in preclinical Alzheimer's disease. *J Alzheimer's Dis* 2016;**52**:77–90. doi:10.3233/JAD-150855.Spatial

15 Miniaci MC, De Leonibus E. Missing the egocentric spatial reference: a blank on the map. *F1000Research* 2018;**7**:168. doi:10.12688/f1000research.13675.1

16 Ritchie K, Carrière I, Su L, *et al.* The midlife cognitive profiles of adults at high risk of late-onset Alzheimer's disease: The PREVENT study. *Alzheimer's Dement* 2017;**13**:1089–97. doi:10.1016/j.jalz.2017.02.008

For peer review only

17 Alexopoulos P, Richter-Schmidinger T, Horn M, *et al.* Hippocampal volume differences between healthy young apolipoprotein e ε2 and ε4 carriers. *J Alzheimer's Dis* 2011;**26**:207–10. doi:10.3233/JAD-2011-110356

18 Ritchie K, Ropacki M, Albala B, *et al.* Recommended cognitive outcomes in preclinical Alzheimer ' s disease : Consensus statement from the European Prevention of Alzheimer ' s Dementia project. *Alzheimer's Dement* 2017;**13**:186–95. doi:10.1016/j.jalz.2016.07.154

19 Fagan AM, Roe CM, Xiong C, *et al.* Cerebrospinal fluid tau/beta-amyloid(42) ratio as a prediction of cognitive decline in nondemented older adults. *Arch Neurol* 2007;**64**:343–9. doi:10.1001/archneur.64.3.noc60123

20 O'Brien JT, Lloyd A, McKeith I, *et al.* A longitudinal study of hippocampal volume, cortisol levels, and cognition in older depressed subjects. *Am J Psychiatry* 2004;**161**:2081–90. doi:10.1176/appi.ajp.161.11.2081

21 Raudys SJ, Jain AK. Small sample size effects in statistical pattern recognition: recommendations for practitioners. IEEE Trans. Pattern Anal. Mach. Intell. 1991;**13**:252–64. doi:10.1109/34.75512

22 Eyben F, Scherer KR, Schuller BW, *et al.* The Geneva Minimalistic Acoustic Parameter Set (GeMAPS) for Voice Research and Affective Computing. *IEEE Trans Affect Comput* 2016;**7**:190–202. doi:10.1109/TAFFC.2015.2457417

23 Anderson AH, Bader M, Bard EG, *et al.* The HCRC map task corpus. 1991;**34**:351–66.

24 Buxó-Lugo A, Toscano JC, Watson DG. Effects of Participant Engagement on Prosodic Prominence. *Discourse Process* 2018;**55**:305–23. doi:10.1080/0163853X.2016.1240742

25 Iaria G, Palermo L, Committeri G, *et al.* Age differences in the formation and use of cognitive maps. *Behav Brain Res* 2009;**196**:187–91. doi:10.1016/j.bbr.2008.08.040

26 ELAN (Version 5.2) [Computer software]. 2018.https://tla.mpi.nl/tools/tla-tools/elan/

27 Sloetjes, H., & Wittenburg P. Annotation by category – ELAN and ISO DCR. In: *Proceedings of the 6th International Conference on Language Resources and Evaluation (LREC 2008)*. 2008.

28 Howes C, Purver M, McCabe R, *et al.* Predicting adherence to treatment for schizophrenia from dialogue transcripts. *Proc 13th Annu Meet Spec Interes Gr Discourse Dialogue* 2012;:79–83.http://dl.acm.org/citation.cfm?id=2392814

29 Howes, McCabe R, Purver M. Using Conversation Topics for Predicting Therapy Outcomes in Schizophrenia. *Biomed Inform Insights* 2013;**6**:39. doi:10.4137/BII.S11661

30 McCabe R, Healey PGT, Priebe S, *et al.* Shared understanding in psychiatrist-patient communication: Association with treatment adherence in schizophrenia. *Patient Educ Couns* 2013;**93**:73–9. doi:10.1016/j.pec.2013.05.015

31 Prud'hommeaux ET, Roark B. Extraction of narrative recall patterns for neuropsychological assessment. In: *INTERSPEECH (Annual Conference of the International Speech Communication Association)*. 2011. 3021–4.

32 Prud'Hommeaux ET, Roark B. Alignment of spoken narratives for automated neuropsychological assessment. *2011 IEEE Work Autom Speech Recognit Understanding, ASRU 2011, Proc* 2011;:484–9. doi:10.1109/ASRU.2011.6163979

33 Roark B, Mithcell M, Hosom J-P, *et al.* Spoken Language Derived Measures for Detecting Mild Cognitive Impairment. *N Engl J Med* 2011;**19**:2081–90. doi:10.1056/NEJMc1108238#SA1

For peer review only

34 García Meilán JJ, Martínez-Sánchez F, Carro J, *et al.* Acoustic Markers Associated with Impairment in Language Processing in Alzheimer's Disease. *Span J Psychol* 2012;**15**:487–94. doi:10.5209/rev_SJOP.2012.v15.n2.38859

35 Satt A, Sorin A, Toledo-Ronen O, *et al.* Evaluation of speech-based protocol for detection of early-stage dementia. *Proc Annu Conf Int Speech Commun Assoc INTERSPEECH* 2013;:1692–6.

36 Lopez-De-Ipiña K, Alonso JB, Solé-Casals J, *et al.* Alzheimer disease diagnosis based on automatic spontaneous speech analysis. In: *Proceedings of the 4th International Joint Conference on Computational Intelligence*. 2012. 698–705.

37 Jones D. A family living with Alzheimer's disease: The communicative challenges. *Dementia* 2015;**14**:555–73. doi:10.1177/1471301213502213

For peer review only

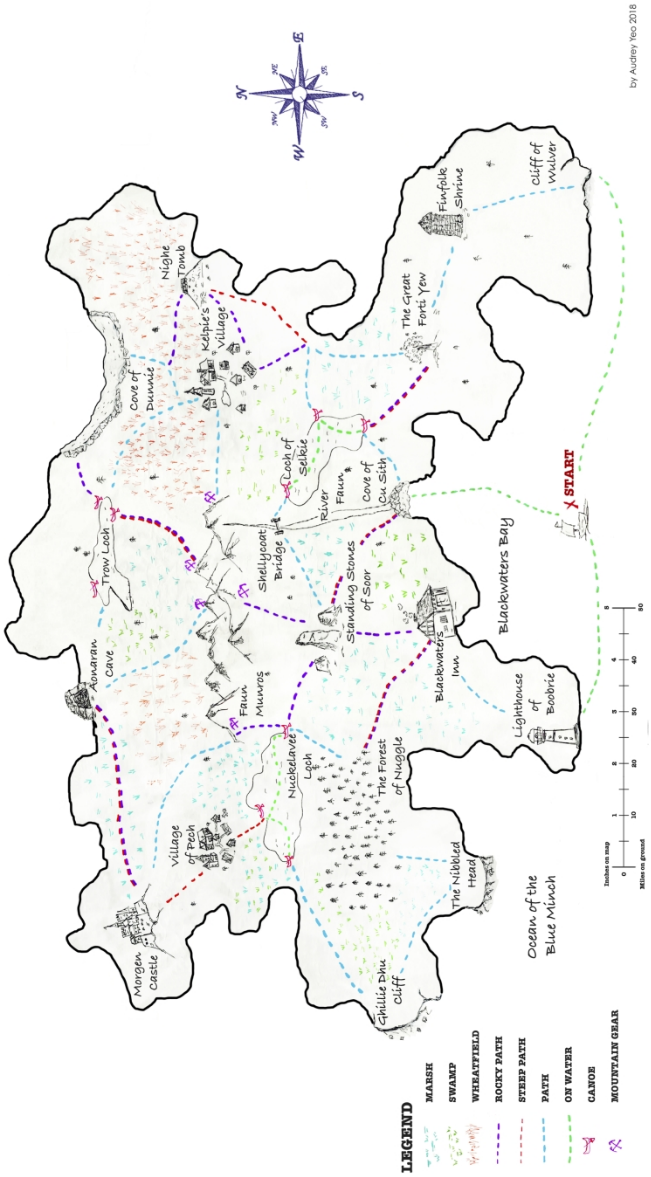

Figure 1. Wayfinding task: map for the participant (with drawn routes)

209x297mm (300 x 300 DPI)

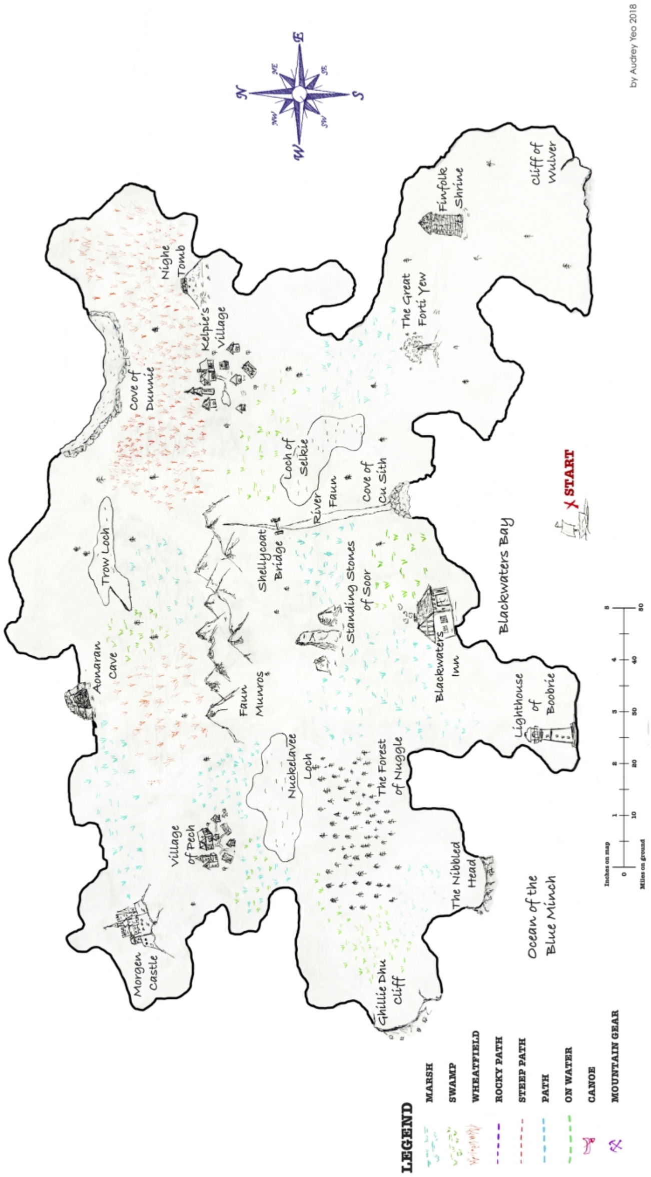

Figure 2. Wayfinding task: map for the researcher (without drawn routes)

209x297mm (300 x 300 DPI)

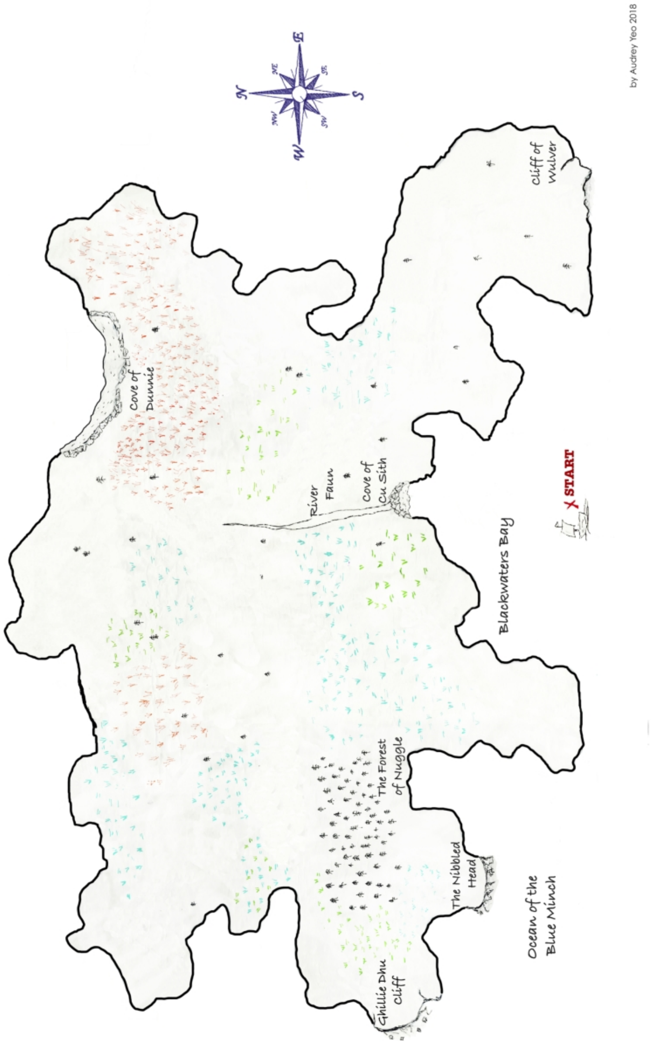

Figure 3. Landmark allocation task: map for the participant (blank version)

209x297mm (300 x 300 DPI)
